# Tumor genotype, location, and malignant potential shape the immunogenicity of primary untreated gastrointestinal stromal tumors

**DOI:** 10.1172/jci.insight.142560

**Published:** 2020-11-19

**Authors:** Daniela Gasparotto, Marta Sbaraglia, Sabrina Rossi, Davide Baldazzi, Monica Brenca, Alessia Mondello, Federica Nardi, Dominga Racanelli, Matilde Cacciatore, Angelo Paolo Dei Tos, Roberta Maestro

**Affiliations:** 1 Unit of Oncogenetics and Functional Oncogenomics, Centro di Riferimento Oncologico di Aviano (CRO Aviano) IRCCS, National Cancer Institute, Aviano, Italy.; 2 Department of Pathology, Azienda Ospedaliera Universitaria di Padova, Padua, Italy.; 3 Department of Pathology and Molecular Genetics, Treviso General Hospital, Treviso, Italy.; 4 Department of Medicine, University of Padua School of Medicine, Padua, Italy.

**Keywords:** Oncology, Cancer, Molecular pathology

## Abstract

Intratumoral immune infiltrate was recently reported in gastrointestinal stromal tumors (GISTs). However, the tumor-intrinsic factors that dictate GIST immunogenicity are still largely undefined. To shed light on this issue, a large cohort (82 samples) of primary untreated GISTs, representative of major clinicopathological variables, was investigated by an integrated immunohistochemical, transcriptomic, and computational approach. Our results indicate that tumor genotype, location, and malignant potential concur to shape the immunogenicity of primary naive GISTs. Immune infiltration was greater in overt GISTs compared with that in lesions with limited malignant potential (miniGISTs), in *KIT/PDGFRA*-mutated tumors compared with that in *KIT/PDGFRA* WT tumors, and in *PDGFRA*-mutated compared with *KIT*-mutated GISTs. Within the *KIT*-mutated subset, a higher degree of immune colonization was detected in the intestine. Immune hot tumors showed expression patterns compatible with a potentially proficient but curbed antigen-specific immunity, hinting at sensitivity to immunomodulatory treatments. Poorly infiltrated GISTs, primarily *KIT/PDGFRA* WT intestinal tumors, showed activation of Hedgehog and WNT/β-catenin immune excluding pathways. This finding discloses a potential therapeutic vulnerability, as the targeting of these pathways might prove effective by both inhibiting pro-oncogenic signals and fostering antitumor immune responses. Finally, an intriguing anticorrelation between immune infiltration and *ANO1/DOG1* expression was observed, suggesting an immunomodulatory activity for anoctamin-1.

## Abstract 2

Integrated immunohistochemical, transcriptomic and computational analysis of gastrointestinal stromal tumors (GIST) reveal that tumor genotype, location, and malignant potential shape immunogenicity.

## Introduction

Gastrointestinal stromal tumors (GISTs) are the most common sarcomas of the gastrointestinal tract (1, 2). The majority of GISTs (~85%) are driven by activating mutations in the gene encoding the receptor tyrosine kinase *KIT* (65%–80%) or *PDGFRA* (15%–20%). The remaining fraction of tumors, overall referred to as *KIT/PDGFRA* WT GISTs (*K/P* WT), may rely on different oncogenic events: activation of the RAS/RAF/MAPK pathway, caused most frequently by *NF1* or *BRAF* mutations (about 10% of the cases); defects in components of the succinate dehydrogenase mitochondrial complex II (SDH) in syndromic gastric GISTs (<5%); and rare (<1%) oncogenic gene fusions (1–6).

Although localized GISTs are potentially curable by surgery alone, a significant fraction of tumors relapses after this treatment. Adjuvant therapy with imatinib targeting activated KIT/PDGFRA proteins proved to be significantly beneficial in the prevention of recurrence and in prolonging the survival of patients with advanced/metastatic disease (1, 7). Yet, some patients are ab initio poorly responsive to this tyrosine kinase inhibitor due to the expression of imatinib-refractory mutations (e.g., *PDGFRA* D842V) or independency of KIT/PDGFRA signaling (*K/P* WT tumors). Moreover, even in responsive patients, imatinib is rarely curative as secondary resistance mutations frequently occur. In these settings, switching to other tyrosine kinase inhibitors, such as sunitinib or regorafenib, has demonstrated clinical benefit (1, 7). Recently, the portfolio of effective drugs used to treat GIST has expanded to also include avapritinib (8) and ripretinib (9).

Mounting evidence indicates that tumor immune microenvironment plays a key role in tumor inception, progression, and response to therapy. In this regard, recent works documented the presence of intratumor immune cell infiltration in GISTs and its effect on imatinib efficacy (10–14). Imatinib has been shown to amplify a preexisting cytotoxic antitumor response by inhibiting tumor cell production of the immune inhibitory enzyme indoleamine 2,3-dioxygenase. In addition, a potentiated effect of imatinib when combined with checkpoint inhibitors (anti-PD1, anti-CTLA-4, or anti-CD40) has been demonstrated in preclinical models (15–17). Based on these preliminary results and on the success of immunomodulatory treatments in other tumor types, several clinical trials aiming at assessing the efficacy of immune checkpoint inhibitors in GISTs are being conducted (NCT01643278, NCT01738139, NCT02500797, NCT02834013, NCT02880020, and NCT03291054) (18–20).

The disclosure of new therapeutic vulnerabilities in GIST is particularly relevant for that fraction of tumors, namely, *K/P* WT GISTs, that are currently orphan of effective therapies. With this in mind, we investigated the immune infiltrate by an integrated immunohistochemical, transcriptomic, and computation approach in what we believe is one of the largest cohorts of primary untreated GISTs analyzed by RNA sequencing to date. Immune contexture was examined in relation to driver gene (*KIT, PDGFRA*, *K/P* WT), tumor location (gastric and intestinal), and malignant potential (miniGIST and overt GIST).

## Results

### In situ evaluation of immune contexture.

As a first step to elucidate the role of immune contexture in GISTs, an explorative cohort of 38 primary untreated GISTs was investigated by IHC. Clinicopathological characteristics of this series are reported in Table 1.

In line with previous studies (12–14), T lymphocytes and macrophages were the most abundant tumor-infiltrating immune cells, in both intestinal and gastric GISTs. CD3^+^ cells ranged between 1 and 117 (median 27.5) per HPF and were distributed as follows (median): CD4^+^ = 4.5, CD8^+^ = 15.0, Foxp3^+^ = 2.0. The number of CD68^+^ cells ranged between 17.0 and 170 (median 53.5). Few CD20^+^ B cells (range 0–19, median 0) and occasional reactivity for PD1 or PDL1 were detected (Supplemental Table 1; supplemental material available online with this article; https://doi.org/10.1172/jci.insight.142560DS1).

**Figure 1:**
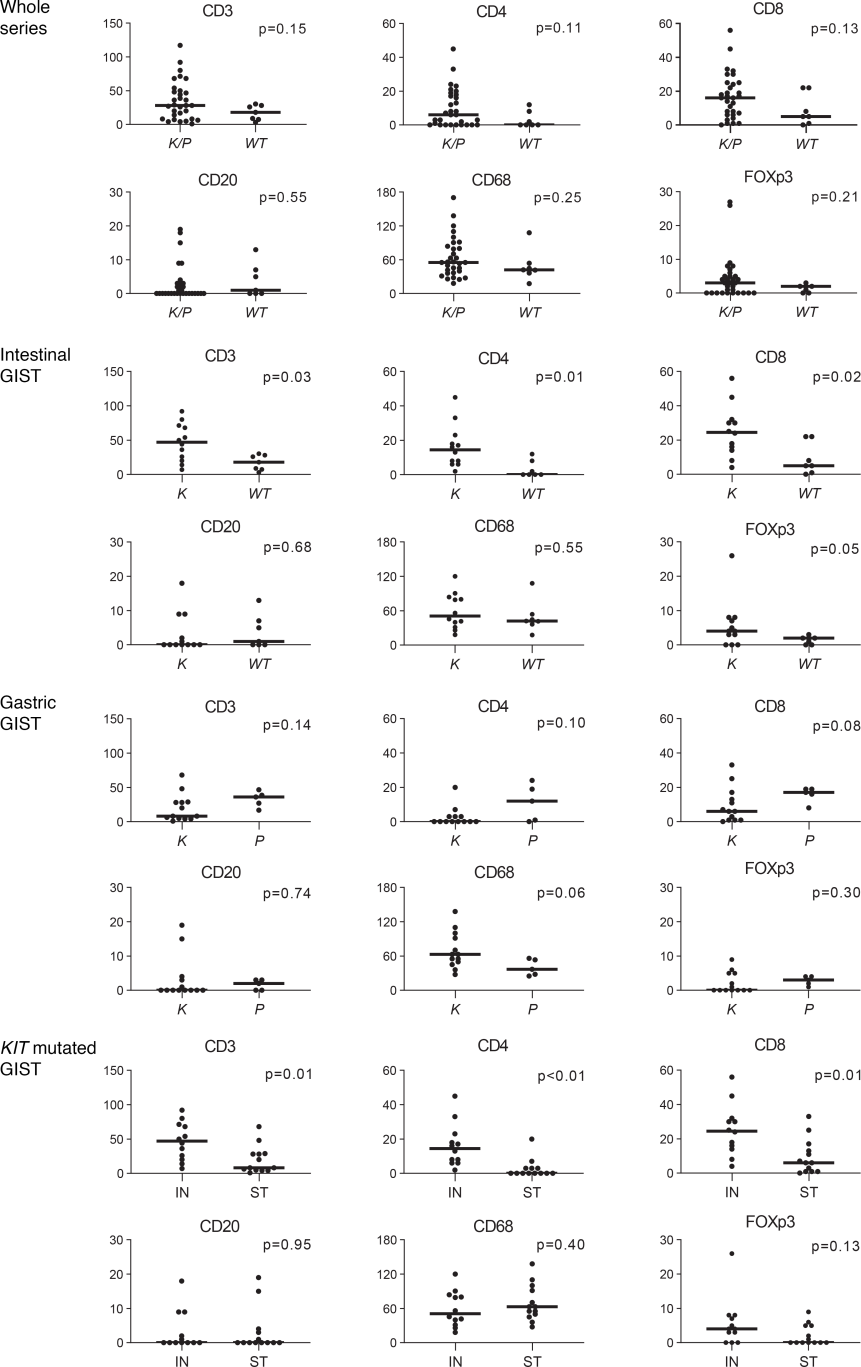
In situ evaluation of immune infiltration in GISTs by IHC analysis. Staining for immune cell markers in the series analyzed as a whole (*n* = 38 cases), and in intestinal (*n* = 19) and gastric (*n* = 18) GISTs analyzed separately. Cases are grouped according to the genotype (K/P, *KIT-*mutated or *PDGFRA-*mutated; WT, WT for *KIT* and *PDGFRA*; K, *KIT*-mutated; P, *PDGFRA*-mutated). The last series of plots shows the positivity for immune cell markers in the cohort of KIT-mutated tumors, grouped according to location (IN, intestine; ST, stomach). The ordinate indicates median number of positive cells per high-powered field. The bar indicates the median value. The Mann-Whitney *U* test was used to compare groups and the *P* value is indicated. GISTs, gastrointestinal stromal tumors.

**Figure 2:**
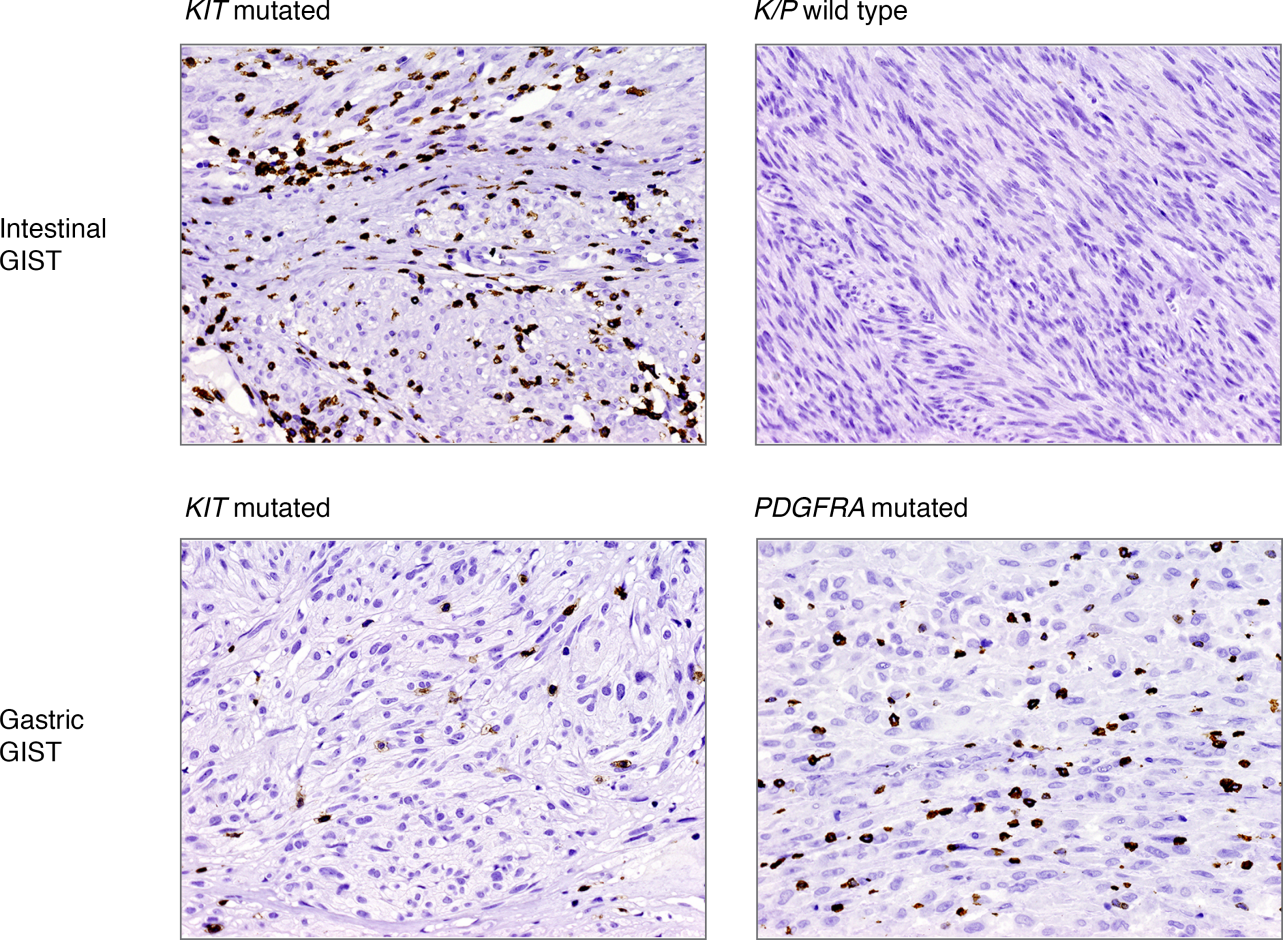
Representative CD3 T cells immunostainings. Location and genotypes are indicated (original magnification, ×20).

When the series was analyzed as a whole, no significant correlation among immune cell types, mitotic index, or tumor site was found. Nevertheless, differences emerged when tumors were compared according to genotype. In particular, the median number of T cells (CD3^+^, CD4^+^, and CD8^+^) was tendentially higher in *K/P*-mutated GISTs than *K/P* WT GISTs (Figure 1 and Figure 2). This difference was particularly evident for the tumors located in the intestine, where it reached statistical significance. Moreover, *KIT*-mutated gastric GISTs featured an inferior degree of infiltration both when compared with *PDGFRA*-mutated gastric tumors and when compared with the *KIT*-mutated counterpart of the intestine (Figure 1).

### Transcriptional assessment of immune infiltration.

To extend this initial observation, we interrogated the transcriptional profile of a cohort of 77 GISTs that were representative of different driver mutations, locations, and malignant potential (Table 1). This series included 33 of 38 cases analyzed by IHC and comprised 62 K/P-mutated tumors and 15 K/P WT tumors (3 *BRAF*, 7 *NF1*-mutated, and 5 WT for all the aforementioned genes as well as for *SDH A-D* genes, and hence defined “driver mutation unknown”) (Table 1).

**Figure 3:**
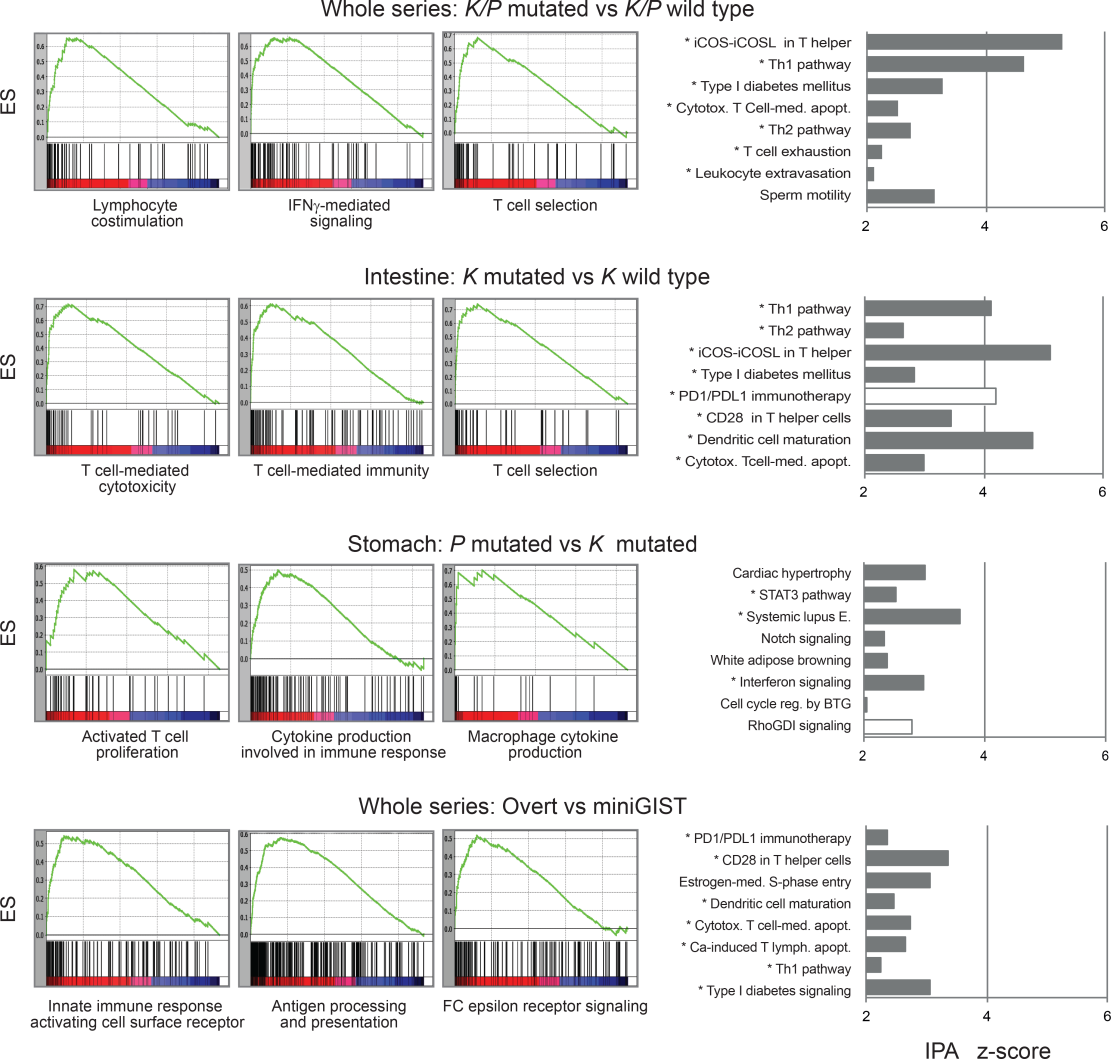
Transcriptional assessment of immune infiltration. The tumor series (*n* = 77 cases) was dichotomized into contrast groups as indicated and the differentially expressed genes were interrogated for immune signatures by using GSEA and IPA. The panels on the left show representative GSEA outputs (GO biological process) with associated ESs. The histograms on the right show the top 8 most significant IPA canonical pathways and associated *z* scores. Pathways strictly related to immunity are indicated by an asterisk. White bars indicate negative *z* scores. GSEA, gene set enrichment analysis; IPA, Ingenuity Pathway Analysis; ESs, enrichment scores.

After the samples were dichotomized into contrast groups according to tumor site (stomach, intestine), malignant potential (miniGIST, overt GIST), and oncogenic driver (*KIT, PDGFRA, K/P* WT), the transcriptome was interrogated for immune signatures by gene set enrichment analysis (GSEA), Ingenuity Pathway Analysis (IPA), and Reactome analyses. Pathways associated with the immune system emerged as significantly enriched in the contrast *K/P*-mutated versus *K/P* WT, particularly in the intestinal subset. A trend for enrichment of immunity-related genes also emerged when contrasting *PDGFRA* versus *KIT* gastric tumors and overt GISTs versus miniGISTs (Figure 3 and Supplemental Table 2). Finally, focusing on *KIT*-mutated GISTs, immunity-related terms were slightly more represented in intestinal tumors than gastric tumors.

These enrichments were paralleled by the differential expression of several immune cell–attracting/activating cytokines, inflammatory interleukins, and related molecules (Supplemental Table 3).

**Figure 4:**
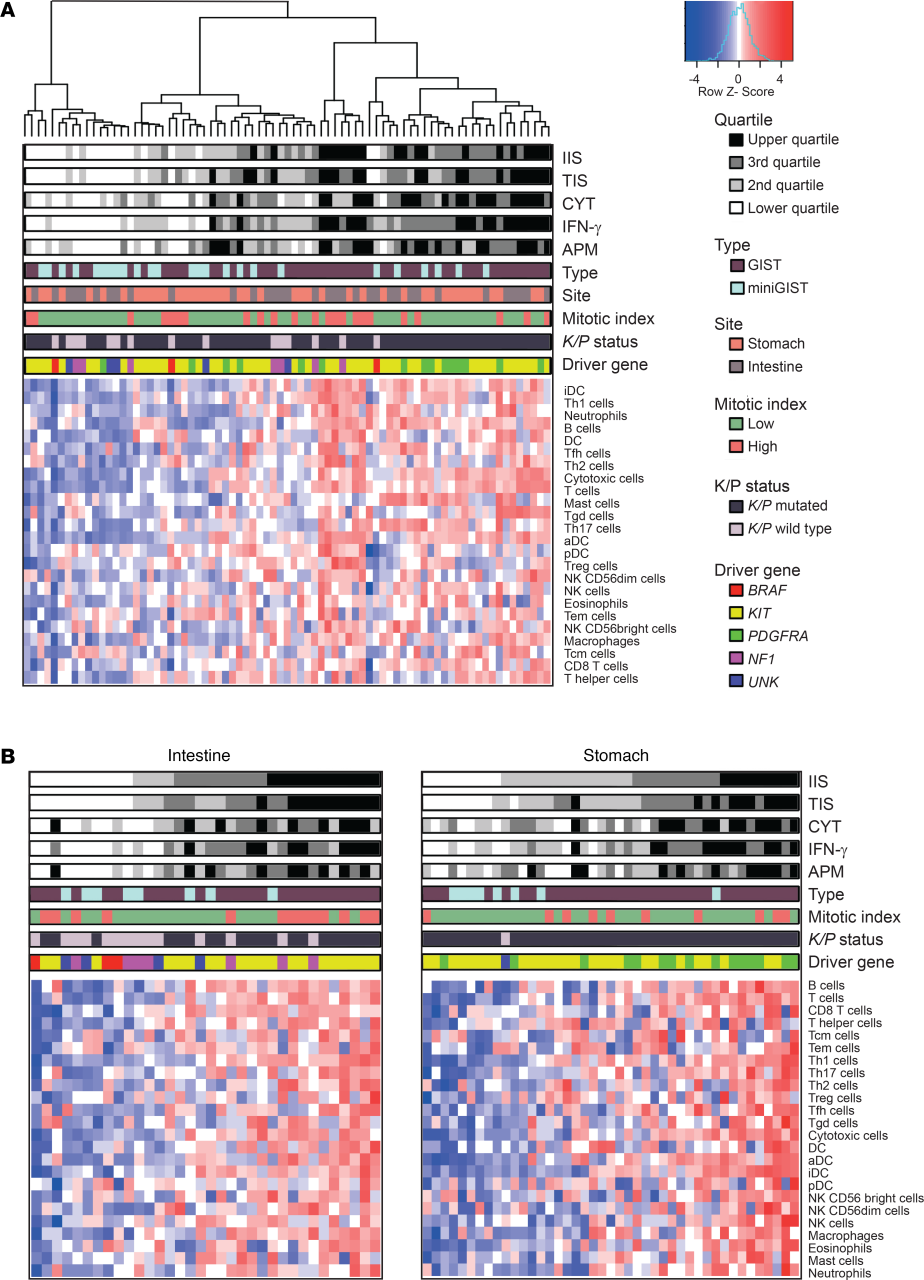
ssGSEA highlights a heterogeneous pattern of immune infiltration in GIST. (**A**) Unsupervised clustering analysis of the whole GIST series (*n* = 77) based on ssGSEA scores of 24 immune cell types. Hierarchical clustering identifies 3 major groups with different extent of immune infiltration. IIS, TIS, CYT, IFN-γ, and APM scores are reported as quartiles. (**B**) ssGSEA in intestinal (*n* = 34) and gastric (*n* = 43) sites analyzed separately highlights the impact of driver gene and malignant potential in immune infiltration. Samples are ordered according to increasing IIS. UNK, driver mutation unknown; ssGSEA, single-sample gene set enrichment analysis; IIS, immune infiltration score; TIS, T cell infiltration score; CYT, cytolytic activity score; APM, antigen-presenting machinery.

To gain better insights into the extent and nature of immune infiltration, several computational methods (single-sample GSEA [ssGSEA], CIBERSORT, and MCP-counter) were exploited to infer the presence of specific immune cell types from bulk transcriptomic data. In particular, ssGSEA was applied to estimate the distribution of 24 immune cell types as well as a cumulative immune infiltration score (IIS) and T cell–specific infiltration score (TIS) per tumor (21). Unsupervised clustering analysis, based on the scores of the 24 immune cell types, identified 3 major groups (Figure 4): the first cluster essentially comprised cold tumors (16 samples) with low IIS and TIS; the second cluster comprised tumors with an “intermediate” degree of infiltration (27 samples); and the third cluster mostly comprised highly infiltrated, “hot” tumors (34 samples).

IIS and TIS were significantly correlated (Spearman’s correlation: *r* = 0.94; *P* = 2 × 10^–7^). Tumors with high IIS were by and large overt GISTs, whereas miniGISTs tended to be cold. A heatmap of the samples ranked according to IIS is provided in Supplemental Figure 1A.

IIS/TIS displayed a positive correlation with the scores for IFN-γ signature (22) (*r* = 0.82, *P* < 1 × 10^–6^) and antigen-presenting machinery (APM) (*r* = 0.63, *P* < 1 × 10^–6^), a proxy for the expression of antigen-processing and presentation molecules. Moreover, IIS, IFN-γ, and APM correlated with the cytolytic activity score (CYT) (23), a surrogate estimate of cytotoxic lymphocyte activation based on the expression of granzyme A and perforin (*r* = 0.62, *r* = 0.83, *r* = 0.82, all *P* < 1 × 10^–6^) (Figure 4A). Overall, these data were suggestive of a potentially proficient antigen-specific immunity in a significant fraction of GISTs.

In line with IHC results on the explorative cohort, ssGSEA indicated that the relative degree of immune infiltration was influenced by tumor genotype. In particular, *K/P*-mutated GISTs featured higher “immunoscores” than *K/P* WT tumors (Mann-Whitney *U* test: IIS, *P* = 0.021; TIS, *P* = 0.006; CYT, *P* = < 0.001) (Supplemental Table 4). Given the intrinsic difference in the biology of gastric and intestinal GISTs (24), we then investigated the immune infiltration correlates for the 2 locations separately (Figure 4B). In the intestine, a higher degree of infiltration was observed in *KIT*-mutated tumors versus *KIT* WT tumors (IIS, *P* = 0.008; TIS, *P* = 0.006; CYT, *P* = 0.001). The same held true for *PDGFRA*-mutated GISTs versus *KIT*-mutated GISTs in the stomach (IIS, *P* = 0.005; TIS, *P* = 0.041; CYT, *P* = 0.004). In both instances, immune infiltration was significantly associated with a greater number of cytotoxic, Th1, Tγδ, and activated dendritic cells as well as higher APM and IFN-γ scores (Supplemental Table 4).

**Figure 5:**
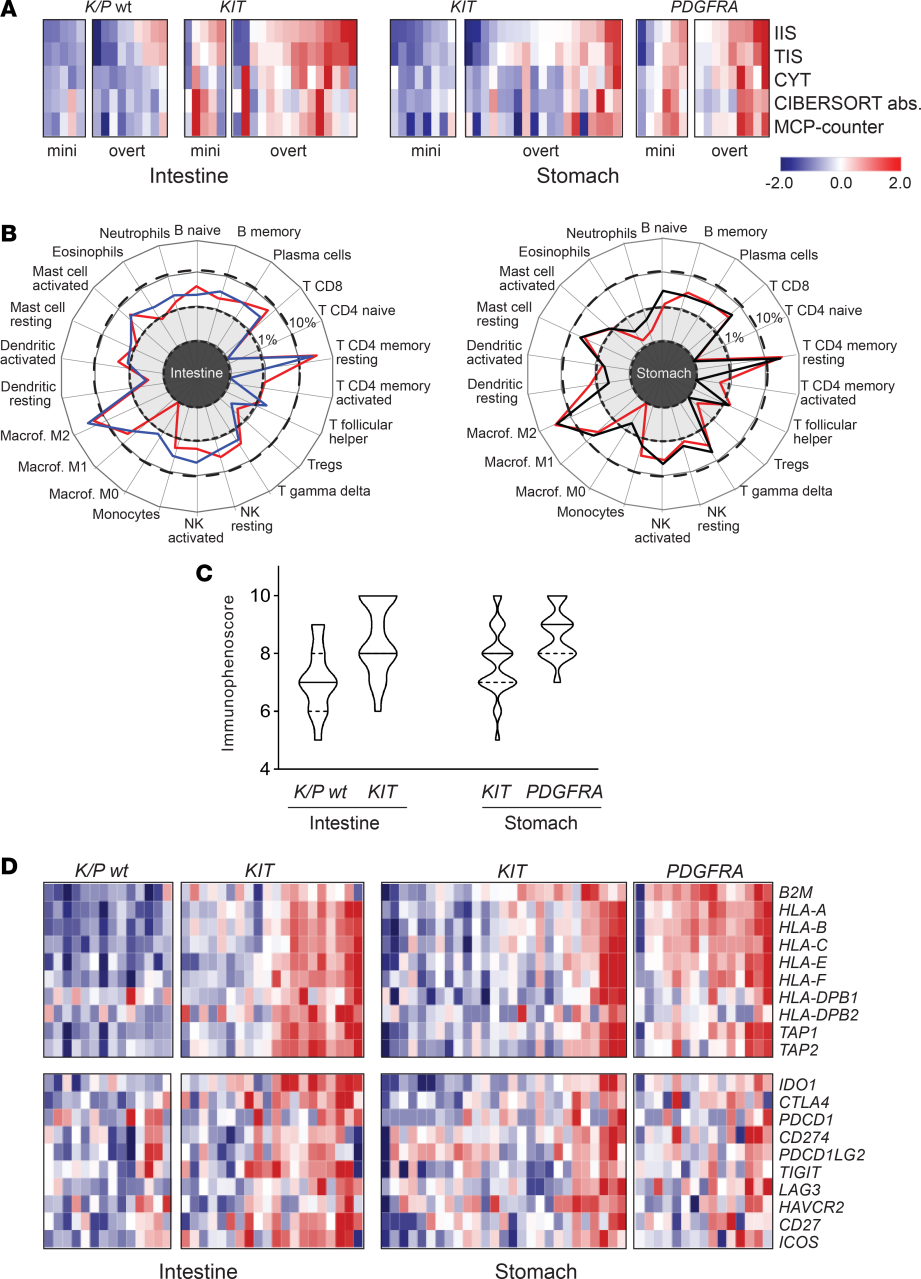
Dissection of genotype, location, and malignant potential in GIST immunogenicity. (**A**) Heatmap of the immune infiltration scores calculated with the indicated algorithms. Color-coded *z* scores for IIS, TIS (ssGSEA), CYT, CIBERSORT absolute (abs), and MCP-counter cumulative scores are shown. Samples are grouped according to tumor location, genotype, and malignant potential (miniGIST and overt GIST). (**B**) Relative proportion of the different immune cell types in intestinal (left) and gastric GIST (right) calculated by CIBERSORT. Mean proportion values (%) of the different cell types were calculated per each genotype (red line, *KIT*-mutated GIST; black line, *PDGFRA*-mutated GIST; blue line, *K/P* WT GIST) and reported in a radar plot. Dotted and dashed lines mark 1% and 10%, respectively. Macrophages M2 and T CD4 memory resting are the most represented immune cell types in both sites. (**C**) Violin plot showing the immunophenoscore of intestinal and gastric GISTs arranged according to genotype. The solid line indicates the median value; dashed lines indicate upper and lower quartiles. (**D**) Heatmap of APM genes and immune modulators in intestinal and gastric GIST. Data are presented as color-coded *z* scores calculated on log2TPM of the whole series (for color coding scale, see **A**). IIS, immune infiltration score; TIS, T cell infiltration score; ssGSEA, single-sample gene set enrichment analysis; CYT, cytolytic activity score; APM, antigen-presenting machinery.

The interrogation of the transcriptome with deconvolution approaches (CIBERSORT and MCP-counter) yielded results coherent with ssGSEA. The cumulative scores obtained with CIBERSORT and MCP-counter showed a trend of colinearity with IIS, TIS, and CYT: all algorithms indicated that *K/P* WT tumors were colder in general compared with *K/P*-mutated GISTs as they were miniGISTs compared with overt GISTs (Figure 5A). Moreover, in line with IHC data, an analysis focused on *KIT*-mutated genotypes highlighted the influence of tumor location on susceptibility to immune infiltration: intestinal *KIT*-mutated tumors featured higher infiltration scores compared with the gastric *KIT*-mutated counterpart (Supplemental Figure 1B and Supplemental Table 4). These patterns were observed in both the whole tumor series, including 64 archival FFPE and 13 frozen specimens, and the sole FFPE subset of samples (64 of 77), indicating that the type of processing did not bias the outcome of these analyses (compare Figure 5A and Supplemental Figure 2). Finally, CIBERSORT, which also estimates the relative proportion of the different immune types within each sample, indicated that M2 macrophages and T cells (particularly CD8 and CD4 memory resting) were the most abundant immune populations, irrespective of tumor site or mutation status (Figure 5B).

Overall, the consistency of RNA-sequencing–based analyses with IHC data supported the robustness of transcriptome-based assessment of immune infiltration and corroborated the notion that tumor genotype, malignant potential, and location impinge upon the GIST immunophenotype.

To address the potential susceptibility to immunomodulatory-based treatments of the different genotypes, we took advantage of immunophenoscore (IPS) (25), a machine learning–based classifier based on the expression of *HLA* genes, immunomodulators, and effector and suppressor cells. This scoring algorithm has proved to be effective in predicting the relative sensitivity to immune checkpoint inhibitors in diverse tumor contexts (25). After having grouped GISTs according to the driver gene, a gradient in IPS scores was observed, with *K/P* WT tumors featuring the lowest IPS values and *PDGFRA*-mutated tumors the highest IPS values (Figure 5C). These trends were supported by a differential expression in *HLA* and immune checkpoint molecules (Figure 5D). Thus, among GISTs, *K/P* WT tumors would be less likely to benefit from immune checkpoint blockade approaches.

### Role of driver mutations as neoantigens.

Given the observed effect of genotype on tumor immunophenotype, we sought to address the theoretical neoantigenic capacity of epitopes generated by the mutated driver gene. Neoantigen prediction algorithms, although far from being precise, may provide hints on the potential binding to patient-matched *HLA* allelotype of peptide sequences spanning the corresponding driver mutation. In this context, NetMHCpan (26) is the one of the most widely used tools. NetMHCpan predicted that almost all mutations yielded at least one peptide capable of binding, with different strengths, a cognate *HLA* allele (Supplemental Table 5).

Because the immunogenic efficacy of a neoantigen is also affected by the extent of expression of the mutated peptide, we compared the expression levels of the driver genes. Whereas *KIT* and *PDGFRA* transcripts were robustly expressed (median TPM: *KIT*, 3239; *PDGFRA*, 2097), *BRAF* was expressed at lower levels (median TPM: 89), which probably influenced its immunogenic power (Supplemental Table 5). Regarding *NF1*, not only was this gene moderately expressed (median TPM 75) but also its alterations were typically frameshift or nonsense mutations that elicited nonsense-mediated mRNA decay. Accordingly, neurofibromin is barely detectable/absent in *NF1*-mutated GISTs (27). Thus, *NF1* mutations are unlikely to yield immunogenic peptides.

### Pathways involved in differential immune colonization.

**Figure 6:**
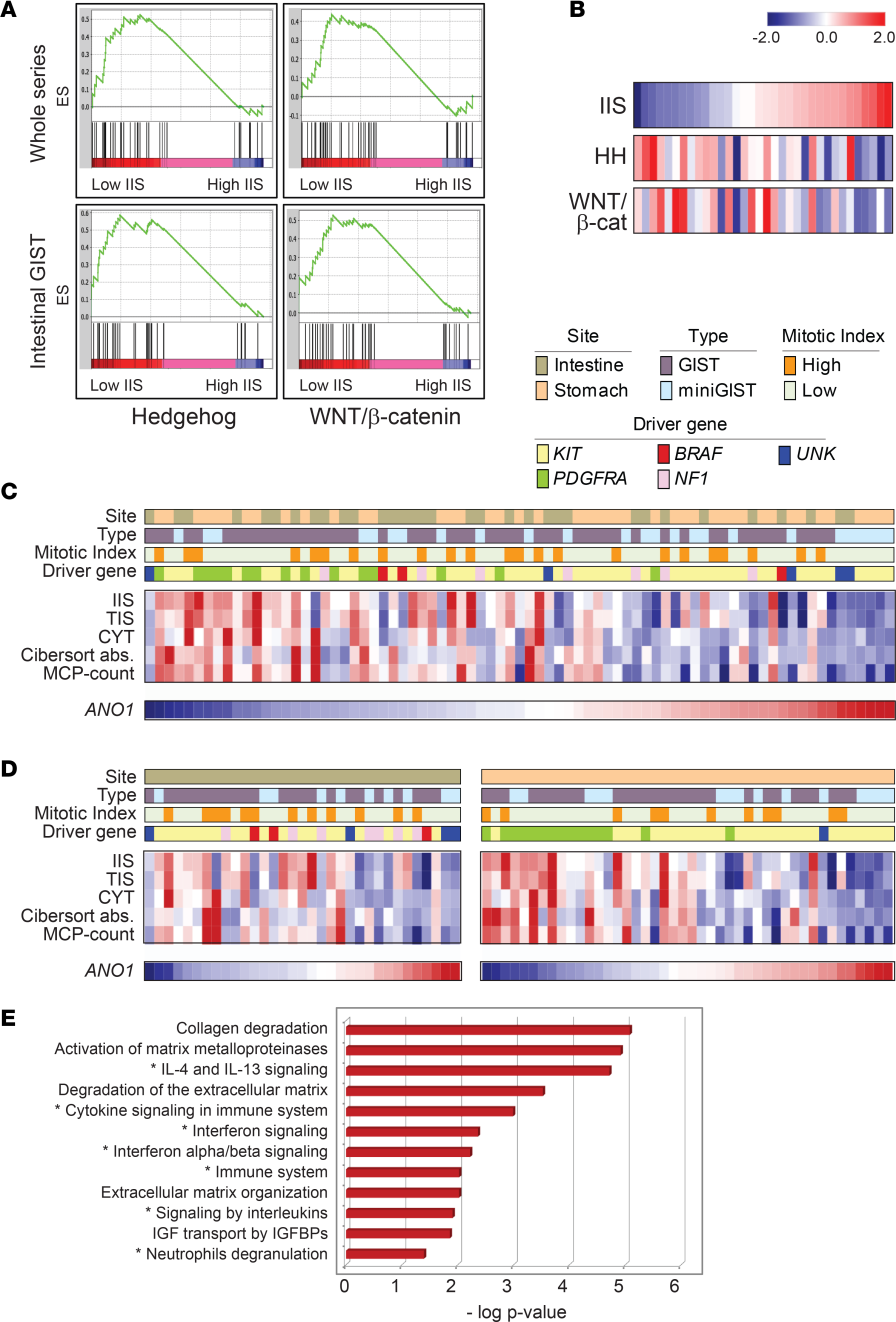
Pathways involved in poorly infiltrated GISTs. (**A**) GSEA analyses indicating the enrichment of HH and WNT/β-catenin MSigDB hallmark signatures in immune cold GIST (low IIS), compared with immune hot GIST (high IIS) in the whole series (top) and in the intestinal subset (bottom). (**B**) Anticorrelation of IIS with HH and WNT/β-catenin activation scores (MSigDB Hallmark) in intestinal GIST. Color-coded *z* score values are displayed. (**C** and **D**) Negative correlation between *ANO1* gene expression and immune infiltration scores in the whole series of 77 cases (**C**) and in intestinal and gastric GIST, separately (**D**). Site, type, mitotic index, and driver gene are as per indicated color-coded labels. *z* Score scale is as in **B**. (**E**) Reactome pathway analysis of the genes differentially expressed following *ANO1* silencing in GIST-T1 cells. The top most statistically significant pathways (–log *P* value, hypergeometric test) are shown. Immune-related pathways are indicated by an asterisk. The input gene list was from ref. 44. UNK, driver mutation unknown; GSEA, gene set enrichment analysis; HH, Hedgehog; IIS, immune infiltration score.

To gain further insights into the mechanisms implicated in shaping GIST immunogenicity, we compared highly and poorly infiltrated tumors by performing GSEA with the MSigDB Hallmark pathway collection. As expected, signaling cascades related to immunity were markedly enriched in high IIS tumors, whereas low IIS tumors featured a trend of enrichment for the Hedgehog (HH) pathway (enrichment score [ES] = 0.52, *P* = 0.05) (Figure 6A and Supplemental Table 6). The enrichment of the HH pathway was particularly evident in poorly infiltrated GISTs located in the intestine (ES = 0.59, *P* = 0.01), where the WNT/β-catenin signaling (WNT/β-cat) also tended to be more represented, although not reaching statistical significance (ES = 0.53; *P* = 0.24) (Figure 6A).

As a complementary approach to address the potential involvement of these pathways in scarcely infiltrated intestinal GISTs, HH and WNT/β-cat pathway activation scores were calculated using both the MSigDB gene sets and 2 other, nonoverlapping HH (28) and WNT/β-cat (29) minimal activation signatures. In both instances, the degree of immune infiltration (IIS) inversely correlated with HH and WNT/β-cat pathway activation scores (HH MSigDB, *r* = –0.53, *P* = 0.02; HH minimal signature, *r* = –0.49, *P* = 0.003; WNT/β-cat MSigDB, *r* = –0.43, *P* = 0.01; WNT/β-cat minimal signature, *r* = –0.36, *P* = 0.038) (Figure 6B and Supplemental Figure 3A).

Furthermore, an RNA-editing event affecting *GLI1*, a major HH downstream target, was detected in a poorly infiltrated *K/P* WT GIST. This rare RNA-editing phenomenon, consisting in an RNA-only nucleotide variation that determines Arg to Gly amino acid change, is known to induce constitutive HH pathway activation (30), thus adding further support to the implication of HH in immune cold GISTs (Supplemental Figure 3B).

HH and WNT/β-cat are highly intertwined signaling routes that have been reported to be associated with phenomena of tumor immune exclusion (31–33). Therefore, the activation of HH and WNT/β-cat pathways might impair GIST immune cell colonization by eliciting immune evasion. Intriguingly, both pathways appear to be positively regulated by the RAS/RAF/MAPK pathway (34–36) and activation of the RAS pathway has also been associated with immune suppression (37, 38). Thus, RAS, HH, and WNT/β-cat might cooperate to dampen the immunogenicity of *K/P* WT intestinal GISTs.

Finally, we were intrigued by the increased expression of *ANO1* (also known as *DOG1* or *TMEM16A*) in poorly infiltrated GISTs (log2FC 0.5; FDR < 0.01). *ANO1*, commonly used as a GIST marker (39, 40), encodes anoctamin-1, an anion exchange molecule that has been recently implicated in chemokine/cytokine secretion (41, 42). We found that *ANO1* inversely correlated with the extent of immune infiltration, an anticorrelation that was particularly evident in the tumors of gastric location (*ANO1*/IIS, whole series: *r* = –0.58, *P* = 3.4 × 10^–8^; stomach: *r* = –0.73, *P* = 2.0 × 10^–7^; intestine: *r* = –0.36, *P* = 0.04) (Figure 6, C and D, and Supplemental Figure 4). The negative correlation between *ANO1* and immune infiltration in GISTs was also confirmed in an independent, publicly available gastric GIST cohort (43) (E-MTAB-373: *ANO1*/IIS, *r* = –0.46, *P* = 0.003).

To gain further insights on the role of *ANO1* in GIST immune colonization, we interrogated the list of genes described as differentially expressed following *ANO1* silencing in GIST-T1 cells (44). Although the limited size of this data set prevents definitive conclusions, overrepresentation analysis indicated enrichment for immune-related signatures (Figure 6E). Overall, these data point to a possible role for anoctamin-1 in modulating tumor immune infiltration.

## Discussion

Recent evidence indicates that tumor-infiltrating immune cells populate the microenvironment of GISTs. A number of IHC studies demonstrated the presence of lymphocytes and macrophages, with some evidence of correlation with disease progression and response to tyrosine kinase inhibitors (10, 11, 13, 14). A broader approach was undertaken by Vitiello and coworkers (12), who combined transcriptional profiling, IHC, and flow cytometry to investigate in deeper detail the immune microenvironment of a large GIST cohort (75 samples). Compared with *KIT*-mutated tumors, *PDGFRA* mutant GISTs were found to feature a greater extent of immune infiltration and cytolytic activity, which were associated with increased levels of chemokines and a greater number of mutation-derived high-affinity neoepitopes. This study primarily focused on gastric tumors, and it included a limited number of small intestinal GISTs (6 of 75) as well as rare genotypes (*NF1* and *BRAF*). Moreover, the series analyzed included both primary and metastatic lesions, naive and treated tumors. Thus, the innate propensity of GISTs to immune infiltration and what tumor-specific factors affect this phenomenon, particularly in uncommon entities such as *K/P* WT tumors, remain to be fully clarified.

Bearing this in mind, we sought to specifically address GIST-intrinsic immunogenicity by focusing on primary imatinib-naive tumors. Our cohort was assembled in a way that the major clinical-pathological and molecular variables affecting GIST biology were well represented. This allowed us to unveil that genotype, location, and malignant potential concur to shape GIST immune contexture.

The presence of intratumor immune infiltrate was demonstrated by integrating immunohistochemical, transcriptomic, and computational approaches. Macrophages and T lymphocytes appeared as the most common infiltrating elements, in line with other studies (11–14, 45).

GISTs with limited malignant potential (miniGISTs) tended to be less infiltrated compared with overt GISTs. This suggests that, in the early phases of development, despite the gain of oncogenic *KIT* or *PDGFRA* mutations, GISTs are relatively immunogenically “silent” and colonization by immune cells somehow accompanies malignant progression.

The role of tumor genotype clearly emerged in both in situ and omics analyses. Specifically, *K/P* WT GISTs turned out to be less infiltrated than *K/P*-mutated tumors.

Several factors probably contribute to the reduced infiltration observed in *K/P* WT tumors. These were primarily intestinal GISTs carrying *BRAF* and *NF1* mutations, and in silico predictions indicated that these mutations were likely had a more limited, if any (see *NF1*-inactivating mutations), neoantigenic potential compared with *KIT/PDGFRA* mutations. In addition, the major dependency of these genotypes on the RAS pathway may play a role in lowering immune colonization. In fact, RAS/RAF/MAPK is the main signaling route in *BRAF* and *NF1*-mutated tumors, whereas in *K/P*-mutated GISTs the activated kinase signals, with variable intensities, through multiple pathways (PI3K/AKT/mTOR, STAT, RAS/RAF/MAPK) (1). The activation of the RAS pathway has been shown to correlate with inhibition of *IFN*γ and *HLA* gene expression, thus lessening lymphocyte infiltration and promoting immune evasion (37, 38, 46).

More interestingly, we found that poorly infiltrated intestinal GISTs featured a peculiar activation of HH and WNT/β-cat pathways. These are 2 highly interconnected and reciprocally regulated pathways. Both intersect the RAS/RAF/MAPK pathway (34, 47) and have been implicated in the pathogenesis of RAS-driven tumors (47–50), including GISTs (51–53). Intriguingly, HH and WNT/β-cat pathways are known to induce immune exclusion: HH suppresses T cell recruitment by inhibiting CXCL9 and CXCL10 production (*CXCL10* was indeed significantly downregulated in *K/P* WT intestinal tumors), and WNT/β-cat activation has been correlated with refractoriness to immune checkpoint blockers (31–33, 54–56).

Taken together, these findings suggest that RAS, HH, and WNT/β-cat likely concur to induce an immune silent phenotype to *K/P* intestinal WT tumors.

In the gastric GIST subset, immune infiltration tended to be greater in *PDGFRA*-mutated tumors compared with *KIT*-mutated tumors, in line with previous findings (12). Higher levels of expression of a set of cytokines that can contribute to the recruitment and activation of immune cells were observed in *PDGFRA*-mutated GISTs. In particular, as reported by Vitiello and coworkers (12), these tumors featured elevated expression levels of *CXCL14*, a cytokine that promotes immune surveillance through recruitment of DC, NK, and CD8 T cells and upregulates *HLA* expression (57). In addition, we observed higher levels of immune-attractant *CCL2* and *CCL4*. CCL2 has a major role in the recruitment of myeloid cells to tumor site and it has been recently implicated in GIST macrophage infiltration (58). Interestingly, PDGFR pathway activation has been shown to induce *CCL2* upregulation in different settings (59–61). The activation of the PDGF pathway has also been shown to induce *IL33* via *SOX7* (62). Accordingly, *SOX7* was overexpressed in *PDGFRA*-mutated versus *KIT*-mutated gastric GISTs, together with *IL33* and *IL15*. Both IL33 and IL15 can potentiate innate or adaptive immune responses by recruiting and stimulating T or NK cells, respectively (63). Thus, the higher level of immune colonization observed in *PDGFRA*-mutated GISTs seem to relate to the activation of the PDGF pathway.

Besides being less infiltrated than the *PDGFRA*-mutated counterpart, *KIT*-mutated gastric GISTs also featured a lower extent of immune infiltration and reduced expression of immunomodulatory cytokines when compared with intestinal GISTs with the same genetic background (*KIT* mutation). This may be due to the specific anatomic microenvironment, but it is also possible that cell-intrinsic factors may be implicated in the differential immune colonization observed in gastric versus intestinal *KIT*-mutated GISTs. In this regard, interstitial cells of Cajal, considered the bona fide cell of origin of GISTs, show distinctive features depending on location, including the expression of cytokines (64).

Finally, an unprecedented finding was the inverse correlation observed between immune infiltration and *ANO1* expression, particularly in the tumors of gastric location. *ANO1*-encoded protein, anoctamin-1, is typically expressed by GISTs, with a diffuse staining pattern generally stronger in *KIT*-mutated and *NF1*-mutated tumors (39, 40). Anoctamin-1 is a calcium-activated anion channel whose chemical inhibition affects GIST cell proliferation and viability (65). Recent evidence also implicates this molecule in chemokine signaling (41). In particular, anoctamin-1 has been shown to suppress the release of proinflammatory cytokines, thus hindering the innate immune response (66, 67). Accordingly, preliminary data suggest that *ANO1* silencing in GIST cells alters the expression of genes involved in immune-related pathways. Overall these data support the notion that anoctamin-1 may play a role in tuning GIST immunogenicity, particularly in the gastric subset.

### What are the clinical implications of this study?

Although imatinib and other tyrosine kinase inhibitors are active in controlling tumor recurrence and progression in patients with advanced disease, still these treatments are hardly curative. Moreover, *K/P* WT tumors currently lack targeted therapies. Our results suggest that a significant fraction of *K/P*-mutated GISTs might benefit from immune-based approaches. Specifically, the evidence of immune colonization by cytotoxic cells and a proficient APM together with the expression of molecules with immune-suppressive functions suggest that immune checkpoint-based therapies may unleash an intrinsic antitumor response in these tumors.

In contrast, *K/P* WT GISTs, in particular *BRAF* and *NF1*-mutated GISTs, were found to be essentially immune silent and hence less likely to benefit from immune checkpoint blockade approaches. The major dependency of these tumors on the RAS pathway may represent a therapeutic opportunity, even in an immunomodulatory perspective. Indeed, the combination of MEK and immune checkpoint inhibitors proved to enhance antitumor immune response in mouse models of RAS-driven cancers (38, 68), and promising results are being achieved in clinical trials with analogous combinations (38).

More interestingly, our study unveiled a therapeutic vulnerability, namely, the implication of HH and WNT/β-cat immune-excluding pathways. In mouse models, chemical inhibition of the HH signaling has been shown to increase the recruitment of cytotoxic cells into tumor and dampen immune-suppressive innate and adaptive response (32). Moreover, combinatorial treatments of immune checkpoint inhibitors with either HH or WNT/β-cat signaling blockade have demonstrated synergistic effects in diverse tumor settings (32, 69–72). Thus, the targeting of HH or WNT/β-cat pathways in poorly infiltrated GISTs, in particular *K/P* WT GISTs, may represent a treatment avenue by both inhibiting intrinsic protumor oncogenic signals and alleviating immune suppression, harnessing the immune system to an antitumor attack.

Finally, the intriguing correlation between *ANO1* expression and degree of immune infiltration points to an additional possible element of vulnerability. Several compounds have demonstrated inhibitory activity toward anoctamin-1, including FDA-approved drugs (65, 73, 74), and chemical inhibition of anoctamin-1 has been shown to affect GIST cell proliferation and survival (65). It would be interesting to evaluate the effect of these compounds on cytokine secretion. Definitively, the implication of *ANO1* in tempering GIST immunogenicity is unprecedented and deserves further investigations.

## Methods

### Samples.

Eighty-two adult cases of primary untreated GISTs were retrieved from the pathology files of the authors’ institutions and reviewed by 3 sarcoma expert pathologists. GIST diagnosis was based on morphology, IHC for CD117 (KIT) and ANO1 (aka DOG1 or TMEM16A), and exclusion of other entities within the differential diagnosis. The series included 57 overt GISTs (≥2 cm; any mitotic index) and 25 miniGISTs, i.e., very low-risk tumors with low mitotic index (≤5 mitoses in 5 mm^2^) and small size (<2 cm). Risk of relapse was calculated according the revised version of Joensuu risk classification (75).

### Mutation analysis.

DNA extraction and mutation analysis were essentially as previously described (4). Briefly, DNA was extracted from tissue sections with a tumor cellularity greater than 70%. Samples were first profiled for *KIT* (exons 9, 11, 13, and 17) and *PDGFRA* (exons 12, 14, and 18) mutations by Sanger sequencing. Samples scoring negative in this analysis were further profiled by using a targeted NGS panel that covered the whole coding sequence of *KIT, PDGFRA, BRAF, NF1, SDH A-D, H/K/N RAS*. The allele frequency of the mutation was greater than or equal to 30%. SDH deficiency was also assessed by SDHB immunostaining.

### IHC analysis of immune infiltrate.

Thirty-eight samples were evaluated for evidence of immune cell infiltration. To this end, samples were stained for CD3, CD4, CD8, FOXp3 (T cells), CD20 (B cells), CD68 (macrophages), and immune checkpoint molecules PD1/PDCD1 and PDL1/CD274. The number of positive cells was determined by counting 15 random high-power fields (HPFs) (×400) in a double-blinded fashion and expressed as median value per HPF. Further details are provided in Supplemental Methods.

### Transcriptome analysis of immune infiltration.

Transcriptional profiling was performed on a series of 77 GISTs, including 64 FFPE and 13 fresh-frozen samples. RNA purification, library preparation, and bioinformatic data analysis are described in detail in Supplemental Methods. Briefly, reads were first checked for quality using FastQC and MultiQC (v1.7) (76). Adapter removal and clipping was done with Trimmomatic (v0.38) (77). Samples reads were aligned against Homo sapiens genome assembly GRCh38 (hg38) with STAR (v2.7.0e) (78). SAMtools (v1.9) (79) was used for merging aligned files. Gene counts were obtained with Cufflinks (v2.2.1) (80). DEseq2 (v3.3) (81) was used for the identification of differentially expressed genes (DEGs).

Pathway analyses were performed on DEGs using IPA (QIAGEN; www.qiagenbioinformatics.com/products/ingenuity-pathway-analysis/) and Reactome (82). GSEA (83) was run on either normalized counts or TPM using Gene Ontology (c5.bp.v7.0) and the MSigDB Hallmark collection of molecular signatures (h.all.v7.0).

The estimate of immune cell infiltration from transcriptome data was performed by using diverse computational methods including ssGSEA (21) and deconvolution approaches, namely, CIBERSORT (84) and MCP-counter (85). IPS (25) and CYT (23) were calculated as previously described.

NetMHCpan (v4.0) (26) was used to predict the binding of *KIT, PDGFRA, BRAF*, and *NF1* mutant peptides to the patient-matched *HLA* class I alleles. PHLAT (v1.0) (86) was employed for determining patient-matched *HLA* alleles. The most common *HLA* alleles in the Italian population (87) were used in 4 cases in which PHLAT typing failed.

HH and WNT/β-cat pathway activation scores were calculated by averaging (geometric mean) log2-transformed TPM values of the genes composing the corresponding MSigDB hallmark signatures (h.all.v7.0) as well as by using 5-gene HH minimal signature reported by Shou et al. (28) and the 16-gene WNT/β-cat signature reported by Chang et al. (29). See Supplemental Methods for further details.

### Data availability.

Raw RNA-sequencing data are accessible at the NCBI-SRA database (https://www.ncbi.nlm.nih.gov/sra, accession PRJNA637476).

### Statistics.

Statistical analyses were performed by SigmaPlot 12.0 (SYSTAT). Correlation coefficients (*r*) were calculated using the Spearman’s rank method. The Mann-Whitney *U* rank-sum test was used to compare groups. Statistical threshold was set at *P* values less than or equal to 0.05.

### Study approval.

The study was performed in compliance with relevant laws and institutional guidelines and was approved by the CRO institutional review board (IRB-04-2017) and by the Marca Ethical Committee (N. 456/CE). Written informed consent was obtained from all patients.

## Author contributions

DG acquired, analyzed, and interpreted the data and drafted and edited the final draft. MS and SR acquired and analyzed the data and provided critical revision of the manuscript. DB provided bioinformatic and statistical data analyses. MB, AM, FN, DR, and MC acquired and analyzed the data. APDT conceived the study and design; analyzed and interpreted the data; acquired funding; and provided critical revision of the manuscript. RM conceived the study and design; supervised the study; analyzed and interpreted the data; acquired funding; and drafted and edited the final draft. All authors read and approved the final version of the manuscript.

## Supplementary Material

supplemental data

supplemental Table 1

supplemental Table 2

supplemental Table 3

supplemental Table 4

supplemental Table 5

supplemental Table 6

## Figures and Tables

**Table 1:**
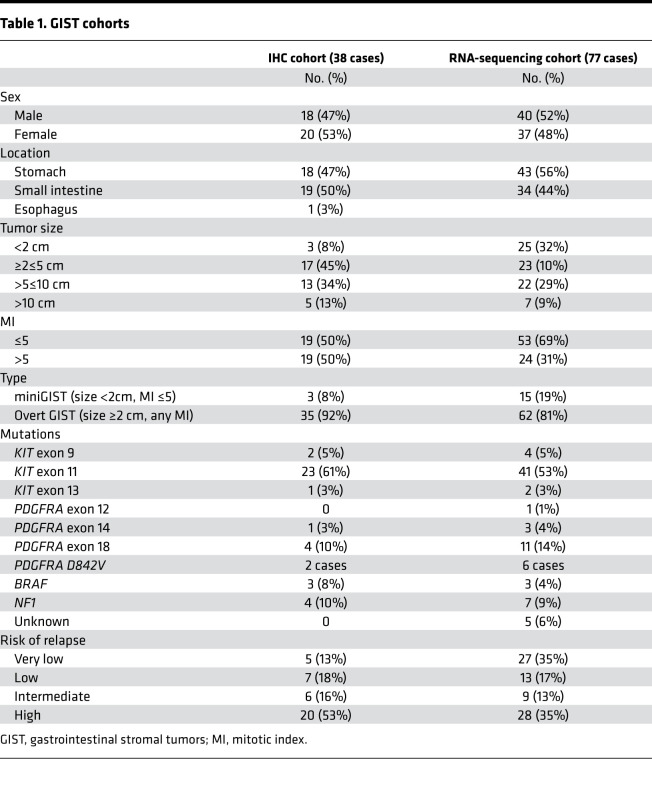
GIST cohorts

## Version 1 10/13/2020

In-Press Preview

## Version 2 11/19/2020

Electronic publication
