# Supplementary material for: Tumor genotype, location, and malignant potential shape the immunogenicity of primary untreated gastrointestinal stromal tumors
Source: JCI Insight. 2020 Nov 19;5(22):e142560. doi: 10.1172/jci.insight.142560 (PMC7710278; doi:10.1172/jci.insight.142560)
Supplement: supplemental data [file jciinsight-5-142560-s096.pdf]

SUPPLEMENTARY MATERIALS AND METHODS

Immunohistochemical analysis of immune infiltrate

Thirty-eight samples were evaluated by IHC for the expression of CD3, CD4, CD8 (T cells), FOXP3 (T-reg cells), CD20 (B cells), CD68 (macrophages), and immune checkpoint molecules PD1/PDCD1 and PDL1/CD274. Immunostains were performed on tissue microarrays where each tumor was represented by three 2-mm cores. The number of positive cells was determined by counting 15 random high power fields (HPF) (400x) in a double-blinded fashion and expressed as median value per HPF.

The following antibodies were used:

CD3: GA503, Rabbit polyclonal (Dako Agilent, Santa Clara, CA); CD4: IR649, Clone 4B12 (Dako Agilent); CD8: GA623, Clone C8/144B (Dako Agilent); FOXP3: ab20034, Clone 236A/E7 (Abcam, Cambridge, UK); CD20: GA604, Clone L26 (Dako Agilent); CD68: GA613, Clone PG-M1 (Dako Agilent); PD1: RA0254-C.5, Clone NAT105 (ScyTek, Logan, UT); PDL1: M3653, Clone 22C3 (Dako Agilent).

Immunostains were performed on tissue microarrays (TMA) where each tumor was represented by three 2-mm cores that were taken from areas located in the central part of the tumor. Tumor margins, peritumoral normal tissues and metastases were not included in the TMA. Necrotic areas, where present, were also excluded.

The counting of IHC positive cells was performed manually, in a blinded fashion, at 400X magnification (high power field, HPF). Per each tumor, 15 random HPFs (5 HPFs per core, along the main axes of the section) were analyzed and median value per HPF calculated (Supplementary Table 1).

RNA sequencing and data pre-processing

Transcriptional profiling was performed on a series of 77 GIST, including 64 Formalin-Fixed Paraffin-Embedded (FFPE) and 13 fresh frozen samples (8 gastric tumors: 6 *KIT* and 2 *PDGFRA* mutated; 5 intestinal tumors: 4 *KIT* and 1 *NF1* mutated). RNA was extracted from tissue sections with tumor cellularity >70%. The Ambion RecoverAll Total Nucleic Acid purification kit (Life Technologies, Carlsbad, CA) was used for FFPE specimens; the EZ1 RNA universal tissue kit (Qiagen, Hilden, Germany) was used for frozen tissues. RNA quality was evaluated with a Bioanalyzer 2100 system (Agilent Technologies, Santa Clara, CA). Libraries were prepared from 0.5-1 microgram of total RNA using the TruSeq Stranded Total RNA Library Preparation kit with Ribo-Zero (Illumina, San Diego, CA). The step of RNA chemical fragmentation was omitted for FFPE samples. Libraries quality and concentration were assessed by using the Agilent 2100 Bioanalyzer DNA 1000 kit (Agilent) and the Qubit DNA HS assay (Life Technologies), respectively. After multiplexing, libraries were run on an Illumina HiSeq1000 system (paired-end, 2x50 cycles). At least 50 million reads were obtained for each sample (median 64 million reads).

Bioinformatics analyses were implemented on Linux systems exploiting online available tools and in-house scripts based on bash, python (v3.6.7) or R (v3.5.0) languages.

FastQC (v0.11.8) (https://www.bioinformatics.babraham.ac.uk/projects/fastqc/) and MultiQC (v1.7) (76) were used to evaluate the quality of raw sequence reads and low quality libraries were excluded from further analyses. Trimmomatic (v0.38) (77) was used to remove adapters and the first 12 residues of each read. Reads were also clipped if, scanning from the 5’ to the 3’ given a sliding window of 15 residues, average quality was below 30.

STAR (v2.7.0e) (78) was used to align reads against Homo Sapiens genome assembly hg38 (GRCh38, Genome Reference Consortium, https://www.ncbi.nlm.nih.gov/grc). Per each sample, aligned files were merged with the Samtools suite of programs (v1.9)(79). Gene counts were obtained with Cufflinks (v2.2.1)(80). Gene coverage, GC content and quality metrics were comparable in libraries generated form

FFPE and frozen samples. The type of sample processing did not represent a major discriminatory factor in the transcriptional profile of our tumor series, as assessed by unsupervised clustering methods. To further rule out the possibility that the results obtained were biased by the type of sample processing, *in silico* analyses of immune infiltration were run both on the whole series of samples (77 cases, 64 FFPE and 13 fresh frozen) and on the sole FFPE subset of cases (See Figure 5A and Supplementary Figure 2). Differential expression analysis was performed by using the DEseq2 pipeline on R (v3.3)(80).

Transcriptome analysis of immune infiltration: GSEA, IPA, ssGSEA, Cytolitic Score, CIBERSORT, MCP-counter and Immunophenoscore

Gene Set Enrichment Analysis (GSEA)(83) was conducted on either DEseq2 normalized counts or TPM using Gene Ontology (c5.bp.v7.0) and the MSigDB Hallmark (h.all.v7.0) gene sets. Basic and advanced parameters were set to default values.

Pathway analyses were performed on differentially expressed genes filtered for |log2fold change|>0.6 and FDR ≤0.1 by using Reactome (82) (https://reactome.org/) and Ingenuity® Pathway core analysis (IPA) (Qiagen).

Single sample GSEA (ssGSEA) was performed over the matrix of FPKM (Fragments per Kilobase Million mapped reads) from all the samples and was run exploiting the R package GSVA (v1.30.0) (https://bioconductor.org/packages/release/bioc/html/GSVA.html). ssGSEA was used to compute a relative quantification of 24 immune cell populations and immune related pathways as described by Şenbabaoğlu *et al* (21). Specifically, by comparing the distribution of gene expression ranks inside and outside a gene set, ssGSEA calculates a sample level gene set score. Cumulative immune infiltration score (IIS) and T-cell specific score (TIS) were calculated as the sum of individual scores of all or T cell specific immune populations. ssGSEA for interferon-γ was performed using the gene signature retrieved from Ayers *et al.* (2017) (22). Unsupervised clustering was performed with Ward linkage and Euclidean distance.

TPM (Transcripts Per kilobase Million mapped reads), quantified by using the CLC Genomics Workbench v12.0 suite (Qiagen), were used for calculating the Cytolitic score (CYT) and for CIBERSORT, MCP-counter and Immunophenoscore analyses.

CYT was calculated by averaging (geometric mean) the TPM values of granzyme A (*GZMA*) and perforin (*PRF1*) per each sample, as described (23).

The CIBERSORT online tool (https://cibersort.stanford.edu/) was used to both obtain relative and absolute quantification of 22 immune cell types. CIBERSORT is a deconvolution approach that relies on a leukocyte gene signature matrix (LM22) and assumes that the overall signature is the result of a weighted sum of the contribution of each cell type-specific signature (84).

The MCP-counter “MCPcounter.estimate” function was used to quantify the absolute abundance of 8 major immune cell types, as described in Becht *et al.* (2016)(85). For each sample a MCP-counter cumulative score was calculated by averaging (geometric mean) the individual scores.

Immunophenoscore was calculated as described in Charoentong *et al.* (2017)(25) by using the dedicated R-script. A cumulative IPS was calculated by averaging the IPS scores of samples grouped according to location and genotype.

Neoantigen prediction

To investigate if the detected *KIT, PDGFRA, BRAF* and *NF1* mutations could act as neoantigens, all the possible peptides spanning the mutation (Mut-peptide) and of lengths comprised between 8 and 11 residues were generated per each sample. In addition, per each patient the Human Leukocyte Antigen *(HLA-A,-B,-C*) gene sequences were determined from transcriptome data by using PHLAT (v1.0)(86).

The binding of Mut-peptides to the patient-matched *HLA* alleles was predicted by using NetMHCpan (v4.0)(26) that provides a score (% rank) that is inversely proportional to the binding strength. NetMHCpan classifies a Mut-peptide as a binder if %rank<2, as a strong binder if %rank<0.5.
In 4 cases patient-specific *HLA* could not be determined. In these cases Mut-peptides were tested against the 20 most common *HLA* alleles in the italian population (87) and the best scoring match recorded.

HH and WNT/β-catenin pathway activation

To identify pathways enriched in poorly infiltrated GIST, IIS scores were used to dichotomize the series into low IIS (negative z-score) and high IIS (positive z-score) samples. GSEA was run with the MsigDB Hallmark collection of molecular signatures (h.all.v7.0). Basic and advanced parameters were set to default values.
Hedgehog and WNT/β-catenin pathway activation scores were obtained by averaging (geometric mean) log2-transformed TPM values of the genes composing each MSigDB Hallmark signature. As a complementary approach, Hedgehog and WNT/β-catenin pathway activation scores were also calculated by using the 5-gene HH minimal signature reported by Shou *et al.* (28) and the 16-gene WNT/β-catenin minimal signature reported by Chang *et al.* (29). These signatures show no overlap with the corresponding MSigDB Hallmark gene sets but for one gene.

Correlation between ANO1 and immune infiltration

The correlation between *ANO1* (aka *DOG1* or *TMEM16A*) expression levels (log2TPM) and immune infiltration (IIS and CYT) was calculated by Spearman rank test. This correlation was validated also on the E-MTAB-373 GIST dataset (https://www.ebi.ac.uk/arrayexpress/experiments/E-MTAB-373) (43).

To gain insights into the pathways affected by *ANO1* in GIST, we performed Pathway Analysis of the 40 genes reported as differentially expressed in GIST-T1 cells following *ANO1* silencing (44).

Statistical analyses

The embedded statistics included in each package was used for bioinformatics-related statistical analyses. Additional statistical analyses were performed by SigmaPlot 12.0 (SYSTAT, San Jose, CA, USA). Correlation coefficients (r) were calculated using the Spearman rank method. The Mann-Whitney rank sum test was used to compare groups. Statistical threshold was set at $p \leq 0.05$.

LEGENDS TO SUPPLEMENTARY FIGURES AND TABLES

Supplementary Figure 1

ssGSEA output of the samples ordered according to increasing Immune Infiltration Score (IIS)

ssGSEA results in the whole series of 77 samples (A) and in the subset of KIT mutated GIST (47 samples), including both gastric (n=27) and intestinal (n=20) tumors (B). Color coded row z-score values are shown.

Supplementary Figure 2

Heat map of the immune infiltration scores in the FFPE subset of samples

Color-coded z-scores for IIS, TIS (ssGSEA), Cytolitic activity (CYT), CIBERSORT absolute (abs) and MCP-counter cumulative scores are shown. Samples are grouped according to tumor location, genotype and malignant potential (miniGIST and overt GIST).

Compared to Figure 5A, this figures shows that that the pattern observed in the whole tumor series (77 GIST including 64 FFPE plus 13 fresh frozen samples) is confirmed also when the analysis is restricted to the group of FFPE samples (n=64) homogeneous for sample processing.

Supplementary Figure 3

Hedgehog and WNT/β-catenin activation in intestinal GIST

A) Intestinal GIST: anticorrelation of IIS with HH and WNT/β-catenin activation scores calculated by using the 5-gene HH minimal signature reported by Shou *et al.* (28) and the 16-gene WNT/β-catenin minimal signature reported by Chang *et a*l (29), respectively.

B) RNA editing of *GLI1* detected in a *K/P* wild type intestinal GIST. A RNA editing event is an ezymatically-induced nucleotide change in the RNA but absent in corresponding DNA sequence. This RNA-only nucleotide variation, detected in a *K/P* wild type GIST, produces an Arg to Gly amino acid change at codon 701 of *GLI1*. This amino acid variation is known to induce constitutive Hedgehog pathway activation (Shimokawa *et al*., 2013)(30).

Supplementary Figure 4

Anticorrelation of *ANO1* with IIS

A) *ANO1* relative gene expression in the whole tumor series ordered according to IIS.

B) Correlation of *ANO1* expression with IIS in intestinal (n=34) and gastric (n=43) sites. Cases are grouped according to genotype. Color-coded z-scores are shown.

Supplementary Table 1

In situ (IHC) evaluation of immune infiltrate.

Supplementary Table 2

GSEA, IPA and Reactome pathway analyses.

Supplementary Table 3

Differentially expressed cytokines, interleukins and related molecules.

Supplementary Table 4

ssGSEA by tumor site and driver gene.

Supplementary Table 5

NetMHCpan binding prediction of Mut-peptides to patient-matched HLA alleles. For each patient, the best NetMHCpan score (%rank) was recorded. Gene expression values of the driver gene are expressed as TPM.

Supplementary Table 6

GSEA analysis for low-ISS *vs.* high-IIS GIST.

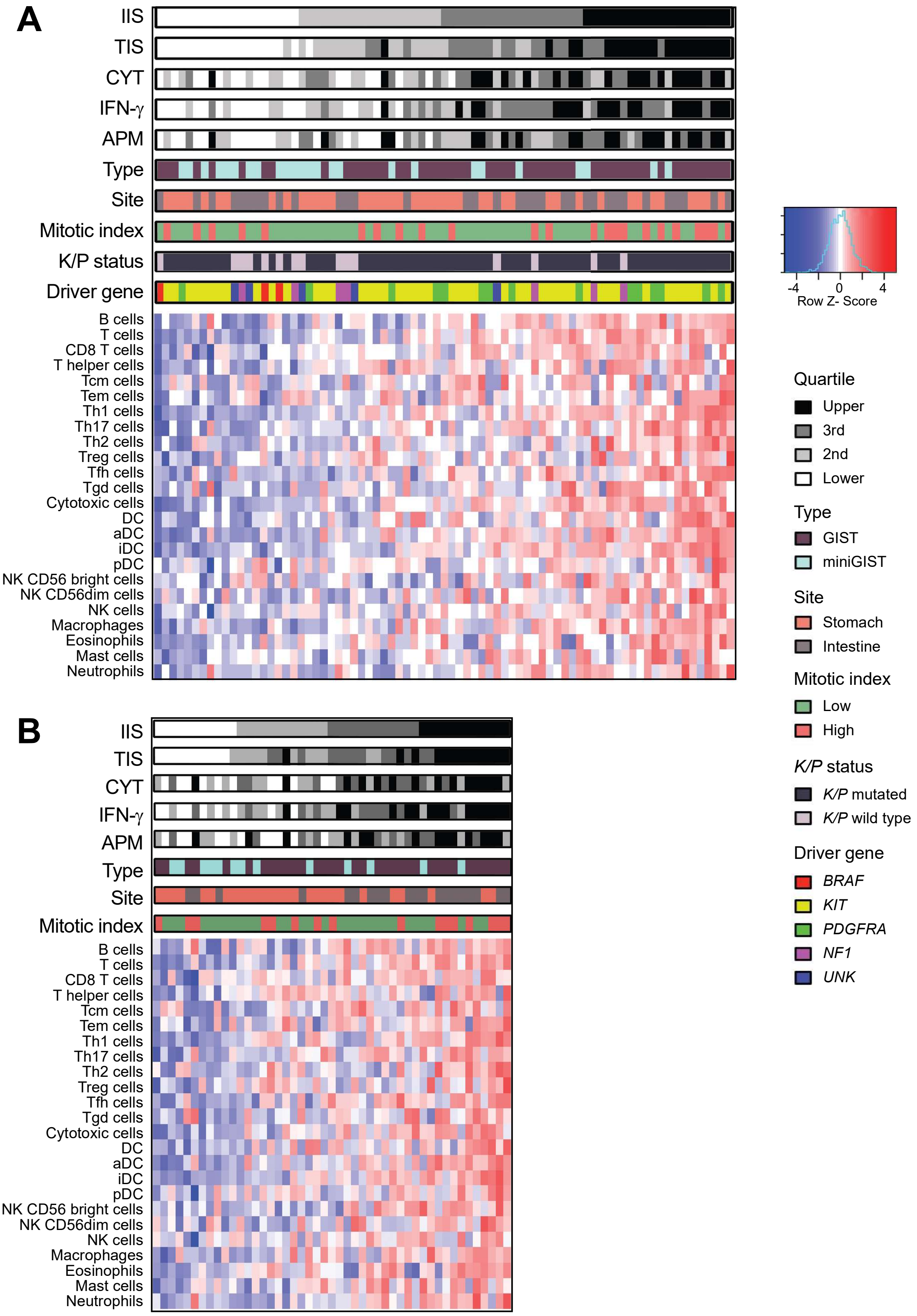

Supplementary Figure 1
ssGSEA output of the samples ordered according to increasing Immune Infiltration score (IIS).
ssGSEA results in the whole tumor series of 77 samples (A) and in the subset of *KIT*-mutated GIST (47 samples) (B), including both gastric (n=27) and intestinal (n=20) tumors. Color-coded row z-score values are shown.

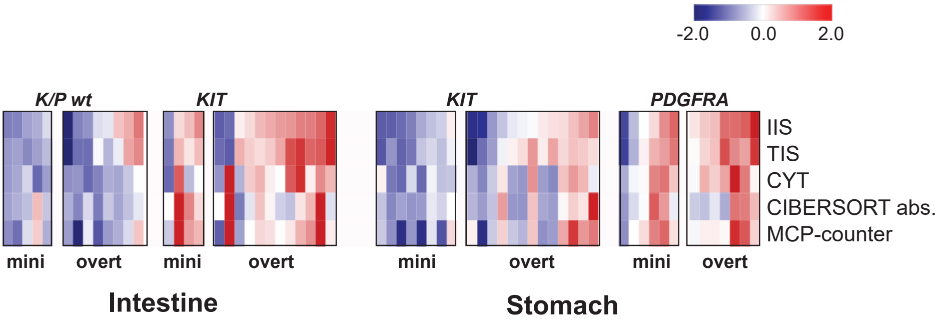

Supplementary Figure 2
Heat map of the immune infiltration scores in the FFPE subset of samples.
Color-coded z-scores for IIS, TIS, Cytolutic activity (CYT), CIBERSORT absolute (abs) and MCP-counter cumulative scores are shown. Samples are grouped according to tumor location, genotype and malignant potential (miniGIST and overt GIST).
Compared to Figure 5A, this figure shows that the pattern observed in the whole tumor series (77 GIST including 64 FFPE plus 13 fresh frozen samples) is confirmed also when the analysis is restricted to the group of FFPE samples (n=64) homogeneous for sample processing.

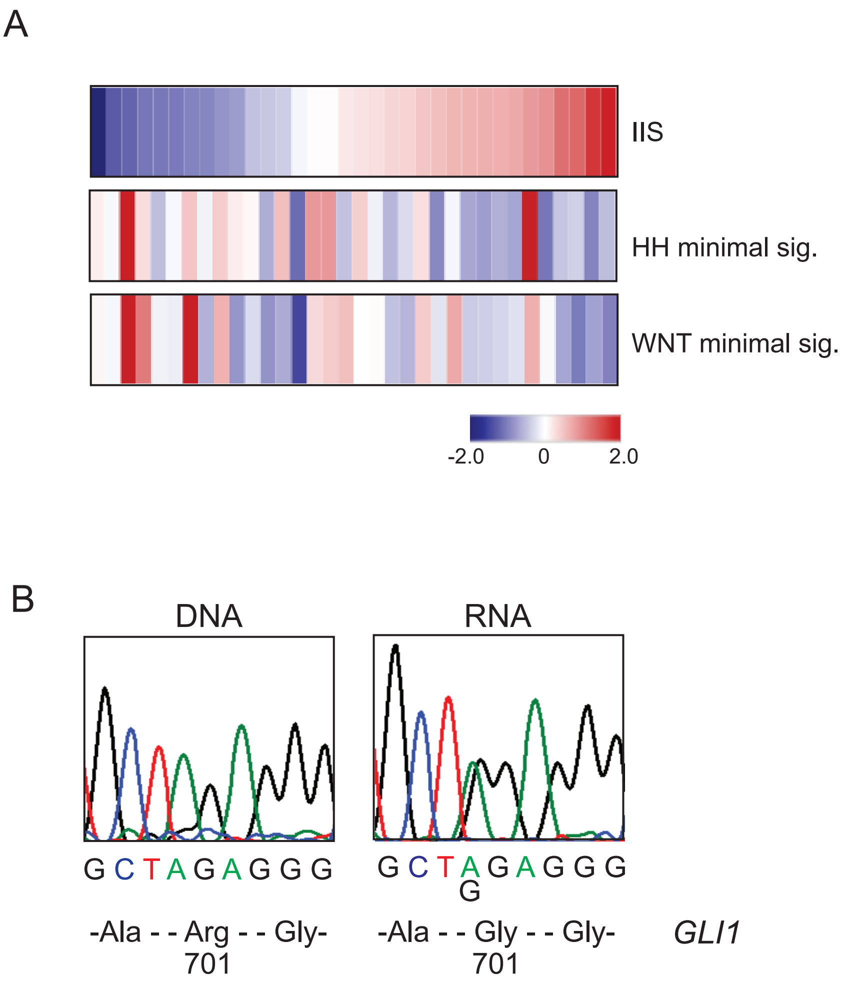

Supplementary Figure 3
Hedgehog and WNT/β-catenin activation in intestinal GIST.
A) Intestinal GIST: anticorrelation of IIS with HH and WNT/β-catenin activation scores calculated by using the 5-gene HH minimal signature reported by Shou *et al.* (28) and the 16-gene WNT/β-catenin minimal signature reported by Chang *et al.* (29), respectively.
B) RNA editing of *GLI1* detected in a *K/P* wild type intestinal GIST. A RNA editing event is an ezymatically-induced nucleotide change in the RNA but absent in corresponding DNA sequence. This RNA-only nucleotide variation, detected in a *K/P* wild type GIST, produces an Arg to Gly amino acid change at codon 701 of *GLI1*. This amino acid variation is known to induce constitutive Hedgehog pathway activation (Shimokawa *et al.*, 2013)(30).

A

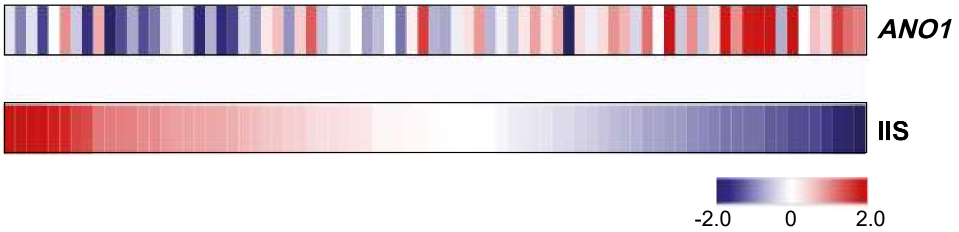

B

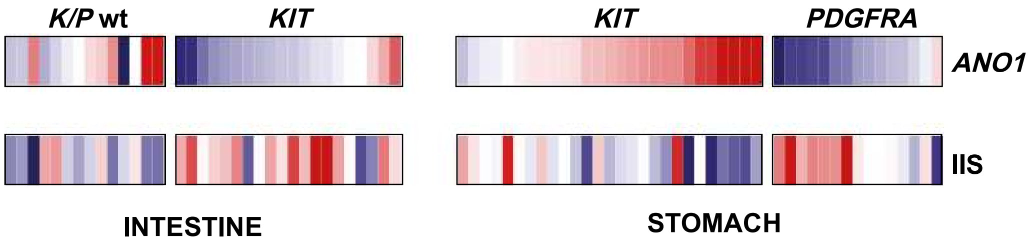

Supplementary Figure 4
Anticorrelation of *ANO1* with IIS
A) *ANO1* relative gene expression in the whole tumor series. Samples are ordered according to IIS.
B) Correlation of *ANO1* expression with IIS in intestinal (n=34) and gastric (n=43) sites. Cases are grouped according to genotype. Color-coded z-scores are shown.
